# (*E*)-1-(2-Bromo­phen­yl)-3-(2,5-dimeth­oxy­phen­yl)prop-2-en-1-one

**DOI:** 10.1107/S1600536810025638

**Published:** 2010-07-07

**Authors:** Jerry P. Jasinski, Ray J. Butcher, K. Veena, B. Narayana, H. S. Yathirajan

**Affiliations:** a Department of Chemistry, Keene State College, 229 Main Street, Keene, NH 03435-2001, USA; b Department of Chemistry, Howard University, 525 College Street NW, Washington, DC 20059, USA; c Department of Studies in Chemistry, Mangalore University, Mangalagangotri 574 199, India; d Department of Studies in Chemistry, University of Mysore, Manasagangotri, Mysore 570 006, India

## Abstract

The title compound, C_17_H_15_BrO_3_, is a chalcone with the 2-bromo­phenyl and 2,5-dimeth­oxy­phenyl rings bonded at opposite ends of a propene group. The dihedral angle between the mean planes of the *ortho*-bromo and *ortho*,*meta*-dimeth­oxy-substituted benzene rings is 77.3 (1)°. The dihedral angles between the mean plane of the prop-2-ene-1-one group and the mean planes of the 2-bromo­phenyl and 2,5-dimeth­oxy­phenyl rings are 58.6 (1) and 30.7 (4)°, respectively. Weak C—H⋯O, C—H⋯Br and π–π stacking inter­molecular inter­actions [centroid–centroid distance = 3.650 (2) Å] are present in the structure.

## Related literature

For the radical quenching properties of included phenol groups, see: Dhar (1981 ▶). For their anti­cancer activity, see Dimmock *et al.* (1999 ▶). For related structures, see: Ng *et al.* (2006 ▶); Rosli *et al.* (2006 ▶). For standard bond lengths, see: Allen *et al.* (1987 ▶).
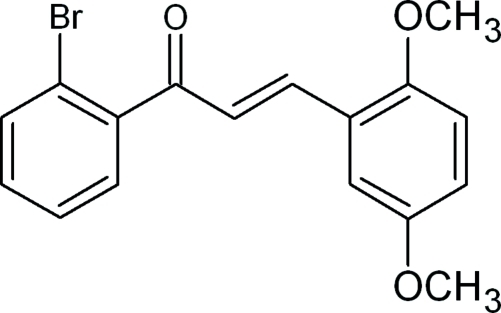


## Experimental

### 

#### Crystal data

C_17_H_15_BrO_3_
*M* *_r_* = 347.20
Triclinic, 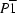

*a* = 7.7643 (9) Å
*b* = 9.7006 (11) Å
*c* = 10.2722 (10) Å
α = 72.901 (10)°
β = 78.487 (9)°
γ = 86.359 (9)°
*V* = 724.59 (14) Å^3^
*Z* = 2
Cu *K*α radiation
μ = 3.93 mm^−1^
*T* = 110 K
0.54 × 0.26 × 0.08 mm

#### Data collection

Oxford Diffraction Xcalibur diffractometer
Absorption correction: analytical (*CrysAlis RED*; Oxford Diffraction, 2007 ▶) *T*_min_ = 0.179, *T*_max_ = 0.635
4759 measured reflections
2835 independent reflections
2749 reflections with *I* > 2σ(*I*)
*R*_int_ = 0.037

#### Refinement

*R*[*F* ^2^ > 2σ(*F* ^2^)] = 0.053
*wR*(*F* ^2^) = 0.146
*S* = 1.05
2835 reflections
192 parameters
H-atom parameters constrained
Δρ_max_ = 2.51 e Å^−3^
Δρ_min_ = −1.11 e Å^−3^

### 

Data collection: *CrysAlis PRO* (Oxford Diffraction, 2007 ▶); cell refinement: *CrysAlis PRO*; data reduction: *CrysAlis PRO*; program(s) used to solve structure: *SHELXS97* (Sheldrick, 2008 ▶); program(s) used to refine structure: *SHELXL97* (Sheldrick, 2008 ▶); molecular graphics: *SHELXTL* (Sheldrick, 2008 ▶); software used to prepare material for publication: *SHELXTL*.

## Supplementary Material

Crystal structure: contains datablocks global, I. DOI: 10.1107/S1600536810025638/fb2201sup1.cif

Structure factors: contains datablocks I. DOI: 10.1107/S1600536810025638/fb2201Isup2.hkl

Additional supplementary materials:  crystallographic information; 3D view; checkCIF report

## Figures and Tables

**Table 1:** Hydrogen-bond geometry (Å, °)

*D*—H⋯*A*	*D*—H	H⋯*A*	*D*⋯*A*	*D*—H⋯*A*
C4—H4⋯Br1^i^	0.95	2.95	3.834 (4)	155
C3—H3⋯O2^ii^	0.95	2.62	3.463 (5)	148

Symmetry codes: (i) 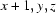; (ii) 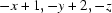.

**Fig. 1.**
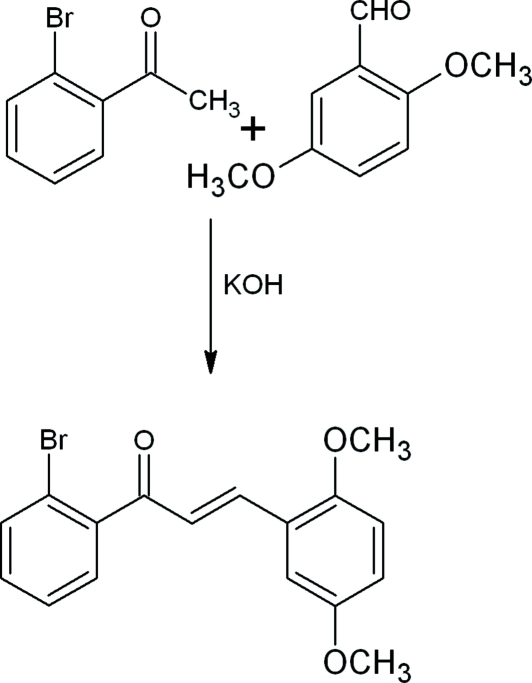
Reaction scheme for C17H15BrO3.

**Fig. 2.**
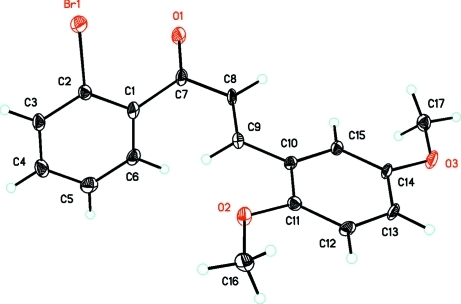
Title molecule, showing the atom labelling scheme with 50% probability displacement ellipsoids.

**Fig. 3.**
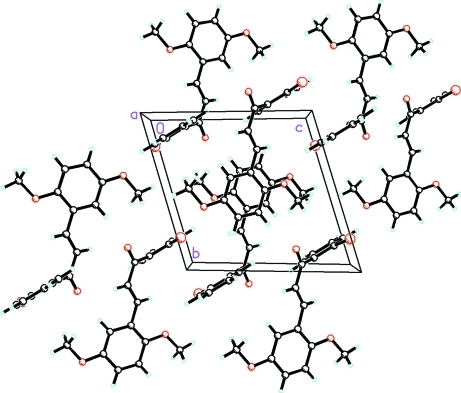
Packing diagram of the title compound, C17H15BrO3 viewed down the a axis. Dashed lines indicate a weak C—H···O intermolecular hydrogen bond interaction.

## supplementary crystallographic information

### Comment

Chalcones, or 1,3-diaryl-2-propen-1-ones, belong to the flavonoid family. Chemically they consist of open-chain flavonoids in which the two aromatic rings are joined by a three-carbon α,β-unsaturated carbonyl system. A vast number of naturally occurring chalcones are polyhydroxylated in the aryl rings. The radical quenching properties of the phenol groups present in many chalcones have raised interest in using the compounds or chalcone rich plant extracts as drugs or food preservatives (Dhar, 1981). Chalcones have been reported to possess many useful biological properties, including anti-inflammatory, antimicrobial, antifungal, antioxidant, cytotoxic, anticancer activities (Dimmock *et al.*, 1999). The crystal structures of closely related chalcones, *viz*., 1-(4-bromophenyl)-3-(2,5-dimethoxyphenyl)prop-2-en-1-one, (Rosli *et al.*, 2006); 1-(4-bromophenyl)-3-(3,4-dimethoxyphenyl)prop-2-en-1-one (Ng *et al.*, 2006) have been reported. Hence in continuation with the synthesis and crystal structure determination and also owing to the importance of these flavanoid analogs, this new bromo chalcone  C_17_H_15_BrO_3_ has been synthesized (Fig. 1) and its crystal structure is reported.

The title compound, C_17_H_15_BrO_3_, is a chalcone with 2-bromophenyl and 2,5-dimethoxyphenyl rings bonded at opposite ends of a propene group (Fig. 2). The dihedral angle between mean planes of the benzene rings in the *ortho*-bromo and *ortho*- *meta*-diimethoxy substituted rings is 77.3 (1)°. The angles between the mean plane of the prop-2-ene-1-one group (C1/C7/O1/C8) and the mean planes of the benzene rings in the 2-bromophenyl (C1–C6)and 2,5-dimethoxyphenyl rings (C10—C15) are 58.6 (1)° and 30.7 (4)°, respectively. Bond distances and angles are in normal ranges (Allen *et al.*,  1987). While no classical hydrogen bonds are present, weak C3—H3···O2, C4—H4···Br1 (Tab. 1) and π-electron ring - π-electron ring interactions [*Cg*1···*Cg*1^i^ = 3.650 (2) Å; i: -*x*, 1 - *y*, 1 - *z*; *Cg*1 is the centroid of the ring C10—C15] are observed which contribute to the stability of crystal packing (Fig. 3).

### Experimental

A 50% (wt) KOH solution (50 ml) was added to a mixture of 2-bromo acetophenone (0.01 mol, 1.99 g) and 2,5-dimethoxy benzaldehyde (0.01 mol, 1.66 g) in 25 ml of ethanol (Fig. 1). The mixture was stirred for an hour at room temperature and the precipitate was collected by filtration and purified by recrystallization from ethanol.  Single crystals (triangular plates with their longest edges 0.3 - 0.5 mm long) were grown from ethyl acetate by slow evaporation method and yield of the compound was 75% (m.p. 355–357 K). Analytical data: Found (Calculated): C %: 58.76 (58.81%); H%: 4.31 (4.35%).

### Refinement

All the hydrogen atoms could have been discerned in the difference electron density map, nevertheless, all the H atoms were constrained in the riding motion approximation. C_aryl_—H = 0.95 Å, with U_ĩso_(H) = 1.20*U*_eq_(C). C_methyl_—H = 0.98 Å, with *U*_iso_(H)=1.50*U*_eq_(C).

### Crystal data

**Table:**

C_17_H_15_BrO_3_	*Z* = 2
*M**_r_* = 347.20	*F*(000) = 352
Triclinic, *P*1	*D*_x_ = 1.591 Mg m^−^^3^
Hall symbol: -P 1	Melting point = 355–357 K
*a* = 7.7643 (9) Å	Cu *K*α radiation, λ = 1.54184 Å
*b* = 9.7006 (11) Å	Cell parameters from 4418 reflections
*c* = 10.2722 (10) Å	θ = 4.6–73.9°
α = 72.901 (10)°	µ = 3.93 mm^−^^1^
β = 78.487 (9)°	*T* = 110 K
γ = 86.359 (9)°	Triangular plate, colorless
*V* = 724.59 (14) Å^3^	0.54 × 0.26 × 0.08 mm

### Data collection

**Table:**

Oxford Diffraction Xcalibur diffractometer	2835 independent reflections
Radiation source: Enhance (Cu) X-ray Source	2749 reflections with *I* > 2σ(*I*)
graphite	*R*_int_ = 0.037
Detector resolution: 10.5081 pixels mm^-1^	θ_max_ = 74.2°, θ_min_ = 4.6°
ω scans	*h* = −9→9
Absorption correction: analytical (*CrysAlis RED*; Oxford Diffraction, 2007)	*k* = −12→11
*T*_min_ = 0.179, *T*_max_ = 0.635	*l* = −12→10
4759 measured reflections	

### Refinement

**Table:**

Refinement on *F*^2^	Primary atom site location: structure-invariant direct methods
Least-squares matrix: full	Secondary atom site location: difference Fourier map
*R*[*F*^2^ > 2σ(*F*^2^)] = 0.053	Hydrogen site location: difference Fourier map
*wR*(*F*^2^) = 0.146	H-atom parameters constrained
*S* = 1.05	*w* = 1/[σ^2^(*F*_o_^2^) + (0.1005*P*)^2^ + 1.9956*P*] where *P* = (*F*_o_^2^ + 2*F*_c_^2^)/3
2835 reflections	(Δ/σ)_max_ = 0.001
192 parameters	Δρ_max_ = 2.51 e Å^−^^3^
0 restraints	Δρ_min_ = −1.11 e Å^−^^3^
58 constraints	

### Special details

**Table:**

Experimental. IR data (KBr) ν cm^-1^: 2832 cm^-1^, 2949 cm^-1^, 2987 cm^-1^ (C—H al. str), 3453 cm^-1^ (C—H ar. str) 1680 cm^-1^ (C=O), 1593 cm^-1^ (C=C); 1231 cm^-1^ (C—O—C).
Geometry. All e.s.d.'s (except the e.s.d. in the dihedral angle between two l.s. planes) are estimated using the full covariance matrix. The cell e.s.d.'s are taken into account individually in the estimation of e.s.d.'s in distances, angles and torsion angles; correlations between e.s.d.'s in cell parameters are only used when they are defined by crystal symmetry. An approximate (isotropic) treatment of cell e.s.d.'s is used for estimating e.s.d.'s involving l.s. planes.
Refinement. Refinement of *F*^2^ against ALL reflections. The weighted *R*-factor *wR* and goodness of fit *S* are based on *F*^2^, conventional *R*-factors *R* are based on *F*, with *F* set to zero for negative *F*^2^. The threshold expression of *F*^2^ > σ(*F*^2^) is used only for calculating *R*-factors(gt) *etc*. and is not relevant to the choice of reflections for refinement. *R*-factors based on *F*^2^ are statistically about twice as large as those based on *F*, and *R*- factors based on ALL data will be even larger.

### Fractional atomic coordinates and isotropic or equivalent isotropic displacement parameters (Å^2^)

**Table:**

	*x*	*y*	*z*	*U*_iso_*/*U*_eq_	
Br1	0.28123 (4)	1.19671 (4)	−0.00743 (4)	0.01849 (18)	
O1	0.1583 (4)	1.1329 (3)	0.3257 (3)	0.0208 (6)	
O2	0.4450 (3)	0.5470 (3)	0.2755 (3)	0.0161 (5)	
O3	0.0011 (4)	0.4011 (3)	0.7884 (3)	0.0177 (5)	
C1	0.4421 (5)	1.0666 (3)	0.2299 (4)	0.0129 (7)	
C2	0.4701 (5)	1.1455 (4)	0.0894 (4)	0.0135 (7)	
C3	0.6379 (5)	1.1881 (4)	0.0155 (4)	0.0160 (7)	
H3	0.6548	1.2397	−0.0802	0.019*	
C4	0.7801 (5)	1.1552 (4)	0.0819 (4)	0.0194 (8)	
H4	0.8945	1.1857	0.0318	0.023*	
C5	0.7563 (5)	1.0772 (4)	0.2223 (4)	0.0203 (8)	
H5	0.8536	1.0560	0.2684	0.024*	
C6	0.5884 (5)	1.0312 (4)	0.2936 (4)	0.0164 (7)	
H6	0.5729	0.9743	0.3878	0.020*	
C7	0.2612 (5)	1.0331 (4)	0.3178 (4)	0.0138 (7)	
C8	0.2125 (5)	0.8849 (4)	0.3976 (4)	0.0138 (7)	
H8	0.1094	0.8697	0.4676	0.017*	
C9	0.3058 (4)	0.7685 (4)	0.3774 (3)	0.0119 (6)	
H9	0.4086	0.7848	0.3070	0.014*	
C10	0.2606 (4)	0.6188 (3)	0.4558 (3)	0.0103 (6)	
C11	0.3385 (4)	0.5059 (4)	0.4034 (3)	0.0116 (6)	
C12	0.3018 (5)	0.3629 (4)	0.4806 (4)	0.0141 (7)	
H12	0.3535	0.2865	0.4454	0.017*	
C13	0.1900 (5)	0.3323 (4)	0.6086 (4)	0.0149 (7)	
H13	0.1676	0.2348	0.6617	0.018*	
C14	0.1098 (5)	0.4435 (4)	0.6606 (4)	0.0128 (7)	
C15	0.1450 (4)	0.5854 (4)	0.5843 (3)	0.0116 (6)	
H15	0.0903	0.6611	0.6193	0.014*	
C16	0.5285 (5)	0.4359 (4)	0.2187 (4)	0.0184 (7)	
H16	0.6043	0.4798	0.1291	0.028*	
H16B	0.4388	0.3772	0.2057	0.028*	
H16C	0.5996	0.3747	0.2825	0.028*	
C17	−0.0777 (5)	0.5153 (4)	0.8422 (4)	0.0174 (7)	
H17	−0.1553	0.4744	0.9317	0.026*	
H17B	−0.1463	0.5775	0.7770	0.026*	
H17C	0.0144	0.5721	0.8548	0.026*	

### Atomic displacement parameters (Å^2^)

**Table:**

	*U*^11^	*U*^22^	*U*^33^	*U*^12^	*U*^13^	*U*^23^
Br1	0.0173 (3)	0.0181 (3)	0.0190 (3)	−0.00149 (16)	−0.00464 (16)	−0.00259 (16)
O1	0.0213 (13)	0.0084 (12)	0.0265 (14)	0.0010 (10)	0.0049 (11)	−0.0020 (10)
O2	0.0204 (13)	0.0086 (11)	0.0171 (12)	−0.0009 (9)	0.0051 (10)	−0.0060 (9)
O3	0.0238 (13)	0.0101 (12)	0.0131 (12)	−0.0010 (10)	0.0029 (10)	0.0021 (9)
C1	0.0176 (17)	0.0045 (14)	0.0157 (16)	−0.0038 (12)	0.0021 (13)	−0.0041 (12)
C2	0.0129 (16)	0.0104 (15)	0.0158 (16)	−0.0017 (12)	−0.0002 (13)	−0.0032 (13)
C3	0.0171 (17)	0.0103 (15)	0.0167 (16)	−0.0030 (13)	0.0031 (13)	−0.0016 (13)
C4	0.0165 (17)	0.0133 (17)	0.0248 (19)	−0.0052 (13)	0.0034 (14)	−0.0038 (14)
C5	0.0188 (18)	0.0118 (16)	0.030 (2)	−0.0014 (13)	−0.0069 (15)	−0.0042 (15)
C6	0.0220 (18)	0.0076 (15)	0.0176 (17)	−0.0005 (13)	−0.0011 (14)	−0.0021 (13)
C7	0.0173 (17)	0.0081 (15)	0.0139 (15)	−0.0034 (12)	0.0015 (13)	−0.0023 (12)
C8	0.0147 (16)	0.0076 (15)	0.0149 (16)	−0.0041 (12)	0.0044 (13)	−0.0007 (12)
C9	0.0127 (15)	0.0114 (16)	0.0106 (15)	−0.0036 (12)	0.0004 (12)	−0.0025 (12)
C10	0.0115 (15)	0.0074 (15)	0.0125 (15)	−0.0011 (11)	−0.0028 (12)	−0.0029 (12)
C11	0.0109 (15)	0.0113 (16)	0.0124 (15)	−0.0002 (12)	−0.0010 (12)	−0.0040 (12)
C12	0.0162 (16)	0.0078 (15)	0.0194 (17)	0.0016 (12)	−0.0041 (13)	−0.0053 (13)
C13	0.0180 (17)	0.0061 (15)	0.0178 (17)	−0.0027 (12)	−0.0046 (13)	0.0022 (12)
C14	0.0145 (16)	0.0090 (15)	0.0117 (15)	−0.0025 (12)	−0.0029 (12)	0.0028 (12)
C15	0.0126 (15)	0.0089 (15)	0.0127 (15)	−0.0013 (12)	−0.0016 (12)	−0.0022 (12)
C16	0.0189 (17)	0.0139 (17)	0.0225 (18)	0.0011 (13)	0.0030 (14)	−0.0107 (14)
C17	0.0183 (17)	0.0167 (17)	0.0140 (16)	−0.0010 (13)	0.0019 (13)	−0.0025 (13)

### Geometric parameters (Å, °)

**Table:**

Br1—C2	1.893 (4)	C8—H8	0.9500
O1—C7	1.227 (4)	C9—C10	1.465 (4)
O2—C11	1.367 (4)	C9—H9	0.9500
O2—C16	1.433 (4)	C10—C15	1.402 (5)
O3—C14	1.375 (4)	C10—C11	1.410 (5)
O3—C17	1.430 (4)	C11—C12	1.396 (5)
C1—C6	1.397 (5)	C12—C13	1.384 (5)
C1—C2	1.401 (5)	C12—H12	0.9500
C1—C7	1.507 (5)	C13—C14	1.399 (5)
C2—C3	1.391 (5)	C13—H13	0.9500
C3—C4	1.383 (6)	C14—C15	1.383 (5)
C3—H3	0.9500	C15—H15	0.9500
C4—C5	1.399 (6)	C16—H16	0.9800
C4—H4	0.9500	C16—H16B	0.9800
C5—C6	1.392 (5)	C16—H16C	0.9800
C5—H5	0.9500	C17—H17	0.9800
C6—H6	0.9500	C17—H17B	0.9800
C7—C8	1.461 (4)	C17—H17C	0.9800
C8—C9	1.348 (5)		
			
C11—O2—C16	117.8 (3)	C15—C10—C9	121.1 (3)
C14—O3—C17	115.6 (3)	C11—C10—C9	119.5 (3)
C6—C1—C2	117.9 (3)	O2—C11—C12	124.4 (3)
C6—C1—C7	118.9 (3)	O2—C11—C10	115.8 (3)
C2—C1—C7	122.8 (3)	C12—C11—C10	119.8 (3)
C3—C2—C1	121.2 (3)	C13—C12—C11	120.0 (3)
C3—C2—Br1	117.5 (3)	C13—C12—H12	120.0
C1—C2—Br1	121.3 (3)	C11—C12—H12	120.0
C4—C3—C2	119.8 (3)	C12—C13—C14	120.7 (3)
C4—C3—H3	120.1	C12—C13—H13	119.6
C2—C3—H3	120.1	C14—C13—H13	119.6
C3—C4—C5	120.4 (3)	O3—C14—C15	124.5 (3)
C3—C4—H4	119.8	O3—C14—C13	115.9 (3)
C5—C4—H4	119.8	C15—C14—C13	119.6 (3)
C6—C5—C4	119.1 (4)	C14—C15—C10	120.6 (3)
C6—C5—H5	120.4	C14—C15—H15	119.7
C4—C5—H5	120.4	C10—C15—H15	119.7
C5—C6—C1	121.5 (3)	O2—C16—H16	109.5
C5—C6—H6	119.2	O2—C16—H16B	109.5
C1—C6—H6	119.2	H16—C16—H16B	109.5
O1—C7—C8	120.6 (3)	O2—C16—H16C	109.5
O1—C7—C1	118.9 (3)	H16—C16—H16C	109.5
C8—C7—C1	120.5 (3)	H16B—C16—H16C	109.5
C9—C8—C7	123.6 (3)	O3—C17—H17	109.5
C9—C8—H8	118.2	O3—C17—H17B	109.5
C7—C8—H8	118.2	H17—C17—H17B	109.5
C8—C9—C10	124.9 (3)	O3—C17—H17C	109.5
C8—C9—H9	117.6	H17—C17—H17C	109.5
C10—C9—H9	117.6	H17B—C17—H17C	109.5
C15—C10—C11	119.3 (3)		
			
C6—C1—C2—C3	−0.4 (5)	C8—C9—C10—C11	163.4 (3)
C7—C1—C2—C3	172.8 (3)	C16—O2—C11—C12	−1.9 (5)
C6—C1—C2—Br1	179.1 (2)	C16—O2—C11—C10	179.4 (3)
C7—C1—C2—Br1	−7.7 (5)	C15—C10—C11—O2	177.9 (3)
C1—C2—C3—C4	−1.4 (5)	C9—C10—C11—O2	−4.0 (5)
Br1—C2—C3—C4	179.1 (3)	C15—C10—C11—C12	−0.9 (5)
C2—C3—C4—C5	1.1 (5)	C9—C10—C11—C12	177.2 (3)
C3—C4—C5—C6	1.1 (6)	O2—C11—C12—C13	−179.1 (3)
C4—C5—C6—C1	−3.0 (5)	C10—C11—C12—C13	−0.4 (5)
C2—C1—C6—C5	2.6 (5)	C11—C12—C13—C14	1.4 (5)
C7—C1—C6—C5	−170.8 (3)	C17—O3—C14—C15	−0.1 (5)
C6—C1—C7—O1	117.6 (4)	C17—O3—C14—C13	178.8 (3)
C2—C1—C7—O1	−55.5 (5)	C12—C13—C14—O3	179.9 (3)
C6—C1—C7—C8	−59.4 (4)	C12—C13—C14—C15	−1.1 (5)
C2—C1—C7—C8	127.4 (4)	O3—C14—C15—C10	178.7 (3)
O1—C7—C8—C9	168.8 (4)	C13—C14—C15—C10	−0.2 (5)
C1—C7—C8—C9	−14.3 (5)	C11—C10—C15—C14	1.2 (5)
C7—C8—C9—C10	179.9 (3)	C9—C10—C15—C14	−176.8 (3)
C8—C9—C10—C15	−18.5 (5)		

### Hydrogen-bond geometry (Å, °)

**Table:**

*D*—H···*A*	*D*—H	H···*A*	*D*···*A*	*D*—H···*A*
C4—H4···Br1^i^	0.95	2.95	3.834 (4)	155
C3—H3···O2^ii^	0.95	2.62	3.463 (5)	148

Symmetry codes: (i) *x*+1, *y*, *z*; (ii) −*x*+1, −*y*+2, −*z*.
